# A cohort study of vitamins contents in human milk from maternal-infant factors

**DOI:** 10.3389/fnut.2022.993066

**Published:** 2022-09-06

**Authors:** Weicang Qiao, Jingyao Chen, Minghui Zhang, Yaling Wang, Baoyu Yang, Junying Zhao, Tiemin Jiang, Lijun Chen

**Affiliations:** ^1^National Engineering Center of Dairy for Maternal and Child Health, Beijing Sanyuan Foods Co. Ltd., Beijing, China; ^2^Beijing Engineering Research Center of Dairy, Beijing Sanyuan Foods Co. Ltd., Beijing, China; ^3^South Asia Branch of National Engineering Center of Dairy for Maternal and Child Health, Guilin University of Technology, Guilin, China

**Keywords:** vitamins, cohort study, dynamics changes, maternal-infant factors, human milk

## Abstract

Human milk vitamin content is an important indicator to evaluate the nutritional composition of human milk. This paper investigates the influence of maternal and infant factors on the dynamics of human milk vitamin content. A total of 147 mother-infant pairs from 3 different cities (north-south distribution) in China were selected and 9 major vitamins were measured in 332 human milk samples. The three vitamins (vitamin A, β-carotene, and pantothenic acid) showed significant downward trends with lactation period (| r | > 0.3, *p* < 0.05). The lactation period factor could explain the negative variation of vitamin A (21.2%) and pantothenic acid (9.5%). The factors of lactation period and oils intake could jointly explain variations of β-carotene (11.8%). (Registration number: NCT02658500).

## Introduction

Vitamins are many different types of low molecular weight organic compounds with different structures and functions. Humans are constantly refining their nutritional needs, and vitamin content has become an important indicator for evaluating nutrition. For infants aged 0–6 months, breast milk is the perfect source of nutrition. The vitamin content of human milk (HM) has a high level of bioavailability (1) even at low concentrations, maintaining the vital activities of infants [metabolism (2, 3), development (4, 5), and antioxidant properties (6, 7)]. Nutritionally, vitamins in human milk can be divided into two categories according to the dissolution method: water–soluble vitamins and fat–soluble vitamins (8). Water–soluble vitamins mainly include vitamin C and B–vitamins. Fat–soluble vitamins mainly include vitamin A (vitamin A and carotenoids), vitamin D, vitamin E, and vitamin K.

Researches (9–13) on vitamins in human milk have focused on developing assay methods and improving storage technology (12), due to the low levels of vitamins in human milk and the varying degrees of loss during collection, storage, transport, and testing of human milk. Studies of prospective mother–infant cohorts are lacking. Most human milk vitamin cohort studies (3, 8, 14, 15) use a cross–sectional study design and data are collected in a retrospective manner, with a lack of prospective follow–up studies based on birth cohorts. This has led to a lack of systematic studies on the effect of maternal and infant factors on the dynamics of vitamin levels. Breastmilk is the gold standard for infant and young children's food (16). However, the individual composition of human milk varies greatly, especially in the evaluation of vitamin nutrition. Due to the large number of vitamin types, a single indicator evaluation cannot better reflect the overall quality of vitamin content in breast milk. Therefore, a comprehensive evaluation of multiple types of vitamins in different human milk is needed to obtain a theoretical analysis model, which is more helpful to provide guidance for the evaluation of vitamins in infant formulae.

Relying on the investigation of Maternal and Infant Nutriomics Cohort Study (MINC) (16), this study selected three cities, Beijing, Luoyang and Liuyang, according to the geographical distribution of China from north to south, to establish a cohort of 147 pairs of prospective mothers and infants exclusively breastfeeding. A total of 147 mother-infant information was collected, including general demographic factors (education and occupation), physiological factors (age, pre-pregnancy BMI, maternal weight gain, infant birth length and infant birth weight), endogenous factors (mode of delivery and infant sex) and exogenous factors (lactation period, geographic location and dietary records). A total of 332 human milk samples were collected from different lactation periods, colostrum (0–5 days of birth), transition milk (12–14 days) and mature milk (1, 3 and 6 months). The relationship between the dynamic changes of vitamins in milk and maternal and infant factors was established by testing the content of nine vitamins in human milk using a multiple linear regression method. This study aims to improve the Chinese healthy breast milk nutrition database in order to fill the gap in the field of human milk vitamin cohort in China and optimize the composition of HM vitamins, improve the quality of human milk, and promote the long- and short-term health of infants.

## Subjects and methods

### Subjects and sampling

This study was an analysis of human milk samples collected from originated from a large mother–infant cohort study (MINC study) in cities China between April 2015 and June 2016. It was approved by the Ethics Committee of Beijing Ditan Hospital affiliated to the Capital Medical University (#2015–027–01) and the study had been registered on Clinicaltrials.gov (registration number: NCT02658500). Human milk samples were collected from healthy lactating women from Beijing (*n* = 146), Luoyang (*n* = 86) and Liuyang (*n* = 100), after the delivery of their full–term infants. Milk samples were collected at 0–5 d (colostrum, *n* = 36), 12–14 d (transitional milk, *n* = 46), 30 ± 2 d (mature milk, 1 month, *n* = 93), 90 ± 2 d (mature milk, 3 months, *n* = 102), and 180 ± 2 d (mature milk, 6 months, *n* = 90). All the samples were placed in a freezing tube. Then within 2 h of sampling, the samples from Beijing were directly delivered to the center stored in the refrigerator at −80 °C by express delivery. The samples from the two other cities were firstly collected to the local cooperating hospitals stored in the refrigerator at −80 °C and sent monthly to the center in Beijing centrally. All samples were tested within one week from the date of receipt. The diet during the first 3 days of sampling were recorded using MultiQuant 3.0.2, a self–developed recording program.

To ensure the accuracy of the data on vitamin content of healthy breast milk in China, the inclusion criteria for healthy mothers and infants were developed in this study with reference to the literature (16). Criteria for a healthy mother and infant: (1) The mother has normal physical indicators on the maternity examination and no chronic diseases (hypertension and diabetes). (2) Absence of immunodeficiency diseases (HIV-infected, cancer, recipients of bone marrow or organ transplants). (3) Exclude those who have received blood transfusion within the last 3 months, those with bleeding disorders, known congenital malformations or genetic defects. Healthy full-term newborns were defined as those within 37–42 weeks of gestational age, with birth weight ≥ 2500 g, and without immunodeficiency disorders and congenital malformations or genetic defects. Referring to the Chinese BMI criteria (16), grouping was done for exclusively breastfeeding mothers.

### Materials

The standards used during the analyses, thiamin (≥ 99.9% purity), riboflavin (≥ 99.9%), niacin (≥ 99.9%), pantothenic acid (≥ 99.9%), vitamin B6 (≥ 99.9%), vitamin C (≥ 98.7%), vitamin A (≥ 99.2%), vitamin E (≥ 99.9%) and β-carotene (≥ 97.6%) were purchased from Sigma–Aldrich (St. Louis, MO, USA). Acetonitrile, formic acid and ammonium formate of LC–MS grade were purchased from Thermo Fisher Scientific (Fair Lawn, NJ, USA). The 0.22 μm syringe filters were purchased from Agela Technologies (Tianjin, China). All other chemicals were of analytical grade and purchased locally. Ultra–pure water was provided by a Siemens Water Technologies system (Warrendale, PA, USA).

### Analysis procedure

#### Water–soluble vitamins

The samples were stored at −80 °C before analysis. The pH of 4 mL human milk was adjusted to 1.7 with 1M HCl then kept in the dark for 2 min. The pH was then adjusted to 4.7 using 1M NaOH and placed in the dark for 2 min. After making up to 10 g with water, the samples were centrifuged and defatted at 10000 × g for 20 min 4 °C (Sigma, Gottingen, Germany). Finally, the supernatants were removed and filtered through a 0.22–μm syringe filter. All samples were prepared in triplicate. Aluminum foil was used to avoid exposing the samples to light for all the following steps. The samples were analyzed by an UltiMate 3000 (Accela)–Q Exactive mass spectrometer (Thermo Fisher Scientific, Waltham, MA, USA) with an ACQUITY UPLC HSS T3 column (HSS T3, 50 mm × 2.1 mm i.d., 1.8 μm) being used for separation. The MS conditions were as follows: source temperature 110 °C, desolvation temperature 350 °C, desolvation gas flow (N_2_) 700 L h^−1^, cone gas flow 30 L h^−1^, and collision gas flow (Ar) 0.13 mL min^−1^. Thermo Scientific Xcalibur software was used for system control and data management.

#### Fat–soluble vitamins

The HM samples were accurately weighed 5 g (to 0.0001 g), and placed in a 50 mL round–bottom centrifuge tube. The HM samples received 5 mL of anhydrous ethanol (99.9%) solution supplemented with vitamin C (0.015 g mL^−1^). Then the samples were added 10 mL of potassium hydroxide solution (1.25 g mL^−1^) and placed in a 55 °C water bath. After saponification for 45 min, the samples were taken out and cooled to room temperature. The saponification solution was added 10 mL ethanol and centrifuged at 1000 × g for 5 min 4 °C. The special solid phase extraction (SPE) cartridges for vitamins were activated by adding 5 mL methanol and 5 mL water. After activation was completed, the saponification liquid supernatant was added. Clean with 10 mL of solution (V_ethanol_: V_water_ = 1:1) and discard all effluent once the supernatant has been thoroughly effused. The SPE column was eluted with 2 mL acetone and 8 mL ethyl acetate after draining for 20 min. The wastewater was collected and dried at 40 °C with nitrogen. Collect all wastewater and dry it at 40 °C using nitrogen. For detection and HPLC analysis, the solution was fixed with 1 mL of methanol and collected over a 0.22–μm microporous membrane.

### Statistics

Data from enrolled mother—infants and human milk vitamins were entered into SPSS 25.0 software for statistical analysis. Normality of the data was verified by the Kolmogorov-Smirnov test. When the data were continuous variables, comparisons between groups were made using the Kruskal-Wallis H test. When the data were categorical variables, between-group comparisons were made using the chi-square test and Fisher's test. All group comparisons were made using the Bonferroni correction. Correlation analysis of vitamin content and mother-infant information was performed using spearman. All data were entered into R and Origin 2021, and heat maps and cluster maps were drawn, respectively. Principal component analysis was used to discriminate between clusters of breast milk vitamin content in different lactations while retaining the maximum information of the original variables.

## Results

### Cohort characteristics

A total of 147 mother-infant pairs were recruited from three cities, Beijing, Luoyang, and Liuyang, in China for this study. Data information of the study population is detailed in Table 1, including general demographic characteristics, physiological characteristics, endogenous characteristics and exogenous characteristics. The distribution of maternal age at childbirth ranged from 18 to 42 years, with a mean of 29 years. The distribution of maternal pre-pregnancy BMI ranged from 15.94 to 30.12 kg/m^2^ with a mean value of 21.65 ± 2.98 kg/m^2^. The population receiving university education accounted for 45.6% of the total. The dietary intake of grain, meat and poultry, eggs, nuts, seeds, soy products and oils of mothers in the three regions exceeded the standards of the dietary guidelines for residents set by the Chinese Nutrition Society. However, the intake of vegetable in Luoyang and Liuyang did not meet the standard, and the intake of seafood in Luoyang did not meet the standard. In terms of fruit and dairy intake, all three regions failed to meet the standard. The proportion of people according to the previous standard the different grades of Pre–BMI was: underweight 20 (13.6%), normal weight 96 (65.3%); overweight 27 (18.4%); obesity 4 (2.7%). Especially in terms of weight gain during pregnancy, more than 54.4% of pregnant mothers achieved strict control of gestational weight gain. Thus, these factors led to a spontaneous delivery rate of 88.4%. The infant cohort included 65 boys and 82 girls, with a weight distribution between 2500 g and 4700 g and a length distribution between 47 and 56 cm.

**Table 1:** Characteristics of the 147 mother-infant pairs and maternal daily dietary intake in China.

	**Range**	***N* = 147**	**Beijing**	**Luoyang**	**Liuyang**	***P*-value**
			***N* = 57**	***N* = 43**	***N* = 47**	
**Age (years)** ^a^	18 ~ 42	29 ± 5	29 ± 5	30 ± 5	27 ± 4	0.024^*^
**Pre-pregnancy BMI** ^a^	15.94 ~ 30.12	21.65 ± 2.98	21.5 ± 2.98	22.42 ± 4.15	21.2 ± 2.68	0.107
**Gestational weight gain, kg** ^a^	0 ~ 49	15.21 ± 6.44	17.07 ± 7.92	13.29 ± 2.86	14.73 ± 5.65	0.027^*^
**Birth weight, g** ^a^	2500 ~ 4700	3412.84 ± 450.7	3443.19 ± 392.57	3548.84 ± 459.62	3251.6 ± 468.18	0.015^*^
**Birth length, cm** ^a^	47 ~ 56	50.07 ± 4.32	50.39 ± 0.92	50.74 ± 1.77	49.06 ± 7.34	0.074
**Education** ^b^						<0.001^**^
Junior high school		37 (25.2%)	5 (8.8%)	19 (44.2%)	13 (27.7%)	
Senior high school		43 (29.3%)	14 (24.6%)	12 (27.9%)	17 (36.2%)	
University education		67 (45.6%)	38 (66.7%)	12 (27.9%)	17 (36.2%)	
**Occupation** ^b^						<0.001^**^
Unemployed		78 (53.1%)	22 (38.6%)	31(72.1%)	24 (51.1%)	
Mental work		36 (24.5%)	22 (38.6%)	8 (18.6%)	8 (17%)	
Physical work		33 (22.4)	13 (22.8%)	4 (9.3%)	15 (31.9%)	
**Gender** ^b^						0.827
Male		65 (44.2%)	27 (47.4%)	18 (41.9%)	20 (42.6%)	
Female		82 (55.8%)	30 (52.6%)	25 (58.1%)	27 (57.4%)	
**Delivery mode** ^b^						0.072
Vaginal		130 (88.4%)	53 (93%)	34 (79.1%)	43 (91.5%)	
Cesarean		17 (11.6%)	4 (7%)	9 (20.9%)	4 (8.5%)	
**Pre-pregnancy BMI (pre-BMI)** ^c^						0.515
Under weight		20 (13.6%)	8 (14%)	4 (9.3%)	8 (17%)	
Normal Weight		96 (65.3%)	39 (68.4%)	26 (60.5%)	31 (66%)	
Over Weight		27 (18.4%)	7 (12.3%)	12 (27.9%)	8 (17%)	
Obese		4 (2.7%)	3 (5.3%)	1 (2.3%)	0	
**Gestational weight gain (WG)** ^b^						0.008^*^
Insufficient		37 (25.2%)	15 (26.3%)	9 (20.9%)	13 (27.7%)	
Normal		43 (29.3%)	8 (14%)	20 (46.5%)	15 (31.9%)	
Excessive		67 (45.6%)	34 (59.6%)	14 (32.6%)	19 (40.4%)	
**Food items (gd** ^**−1**^ **)** ^a^						
Grains		641.68 ± 18.4	746.3 ± 40.2	819.7 ± 69.4	524.4 ± 37.8	<0.001^**^
Vegetable		345.44 ± 18.76	343.3 ± 37.3	260.7 ± 34.4	274.4 ± 33.1	0.654
Fruit		123.28 ± 7.99	147.5 ± 16.3	141.7 ± 36.3	90.1 ± 13.0	0.301
Meat, Poultry		179.97 ± 10.66	174.9 ± 20.3	195.0 ± 43.6	175.0 ± 20.2	0.546
Seafood		138.86 ± 16.86	159.3 ± 36.4	33.0 ± 13.9	74.6 ± 11.8	0.002^*^
Eggs		74 ± 4.78	79.5 ± 9.9	95.8 ± 13.6	55.7 ± 7.3	0.104
Dairy		70.8 ± 6.66	108 ± 13.4	162.3 ± 32.9	35.9 ± 12.7	<0.001^**^
Nuts, Seeds, Soy Products		74.15 ± 7.76	34.8 ± 8.3	35.7 ± 13.4	71.3 ± 15.3	0.004^*^
Oils		7.34 ± 0.62	6.0 ± 1.1	3.8 ± 1.4	9.7 ± 2.0	0.021^*^
**EI (kcal)** ^a^		2195.99 ± 63.73	2291.8 ± 134.9	2733.5 ± 270.6	1787.8 ± 111.9	0.006^*^

Characteristics data of mother-infant pairs are expressed as mean ± SD for continuous variables and number (percentage) for categorical variables. Data of daily dietary intake is expressed as mean ± SE for continuous variables.

* Indicates a significant difference amongst the three groups (P < 0.05).

a Compared by Kruskal-Wallis test.

b Compared by Chi-square test.

c Fisher's precision probability test.

### HM samples

**Figure 1:**
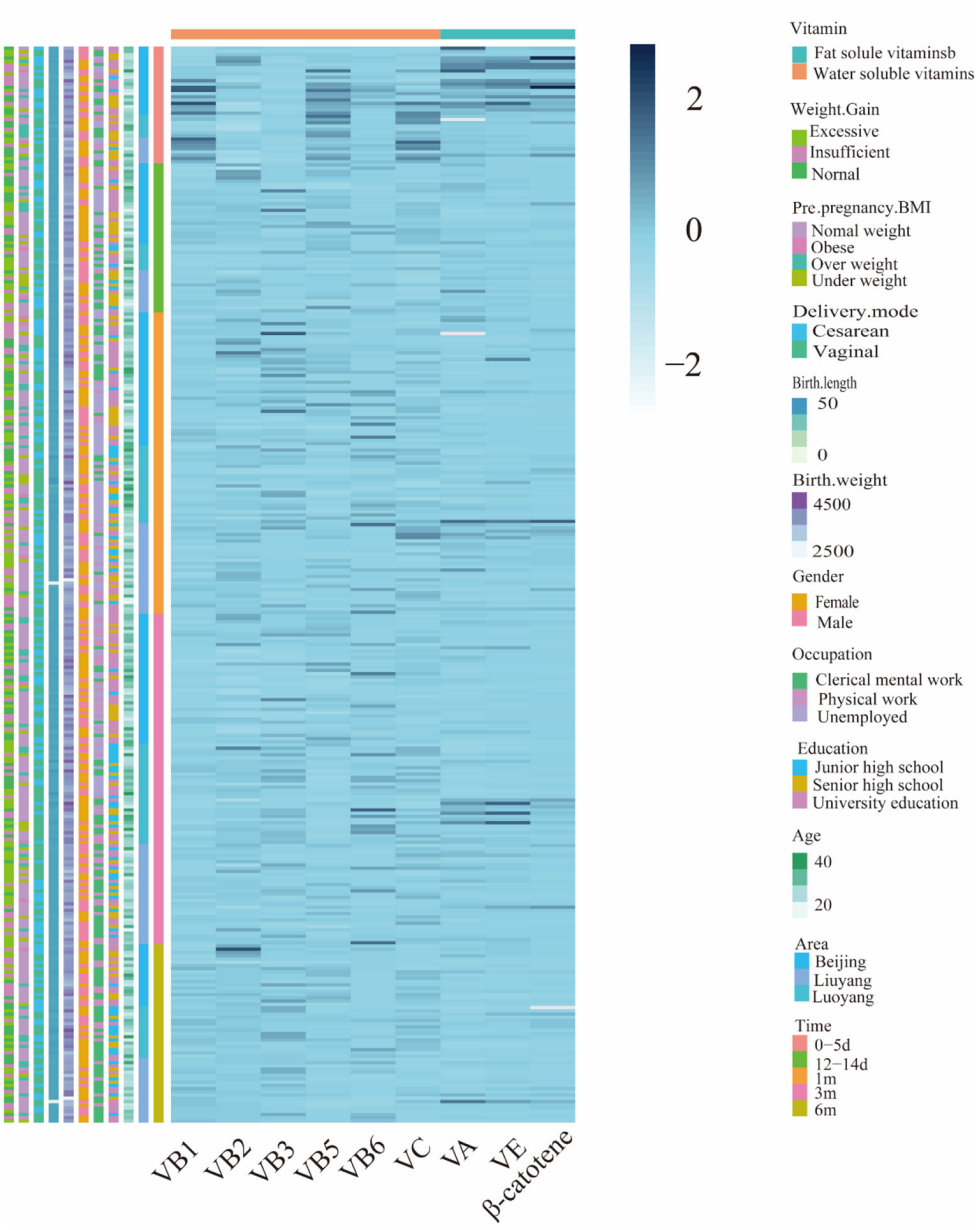
Heatmap displays different classes of vitamin composition (z-scores) for 332 samples in the China MINC study cohort.

**Figure 2:**
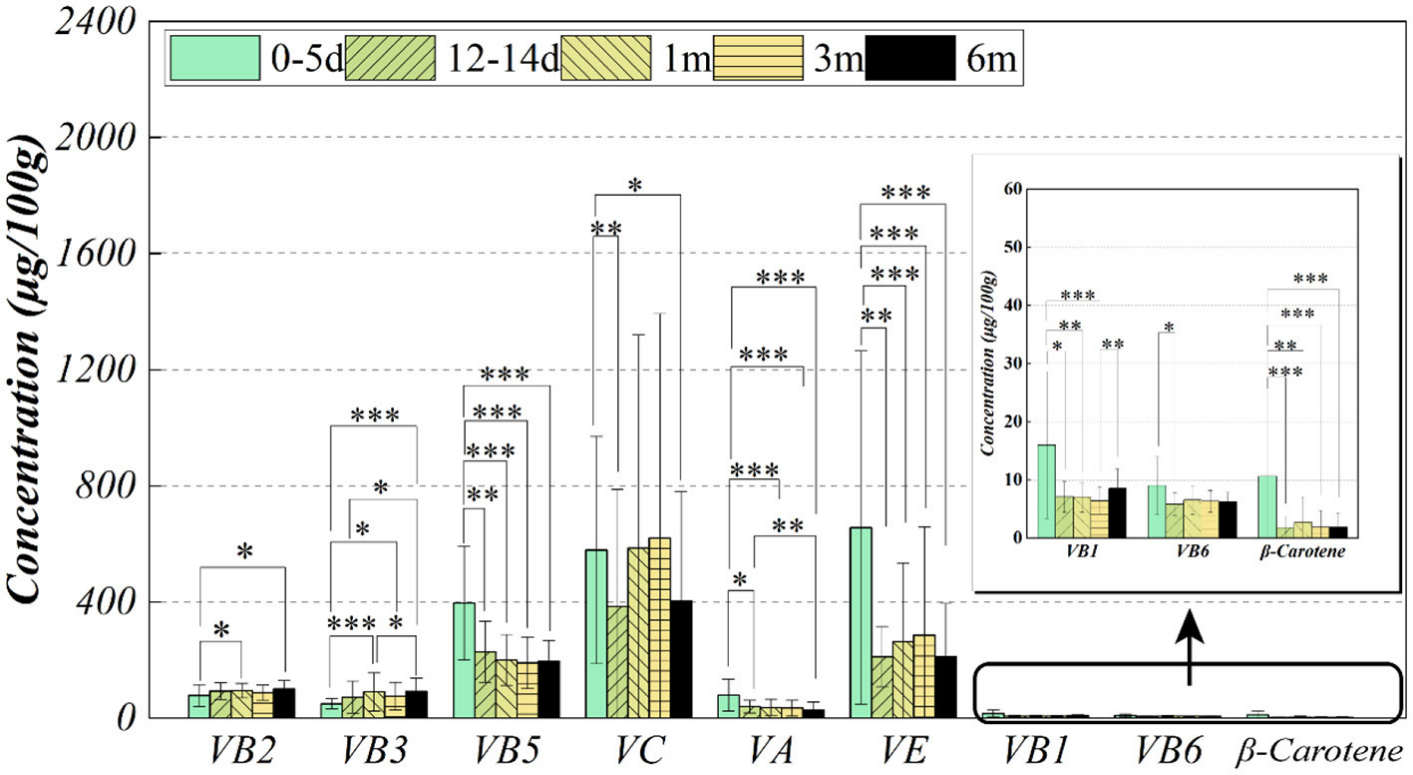
The HM vitamins contents change with lactation period.

Figure 1 showed the cluster visualization heatmap of vitamins (Z–scores) for the samples in the cohort. Each column was clustered by vitamin group, and each row was clustered according to cohort samples information. Because the vitamins showed a positive distribution by K-S verification. As shown Figure 2, the results showed that vitamins contents were highest in colostrum, except for riboflavin, niacin and vitamin C. Krustal–Kalliss H was used to compare the changes of HM vitamins during different lactation periods. Significant differences in comparison between groups were thiamine, niacin, pantothenic acid, vitamin A, β-carotene and vitamin E. The average vitamins contents in HM from 0–6 months were thiamine (8.07 ± 5.59 μg 100g^−1^), riboflavin (91.22 ± 28.89 μg 100g^−1^), niacin (78.96 ± 53.43 μg 100g^−1^), pantothenic acid (221.68 ± 121.45 μg 100g^−1^), vitamin B6 (6.59 ± 2.67 μg 100g^−1^), vitamin C (634.23 ± 537.50 μg 100g^−1^), vitamin A (39.36 ± 34.13 μg 100g^−1^), β-carotene (6.21 ± 3.05 μg 100g^−1^) and vitamin E (354.85 ± 269.68 μg 100g^−1^).

### Correlation analysis

**Figure 3:**
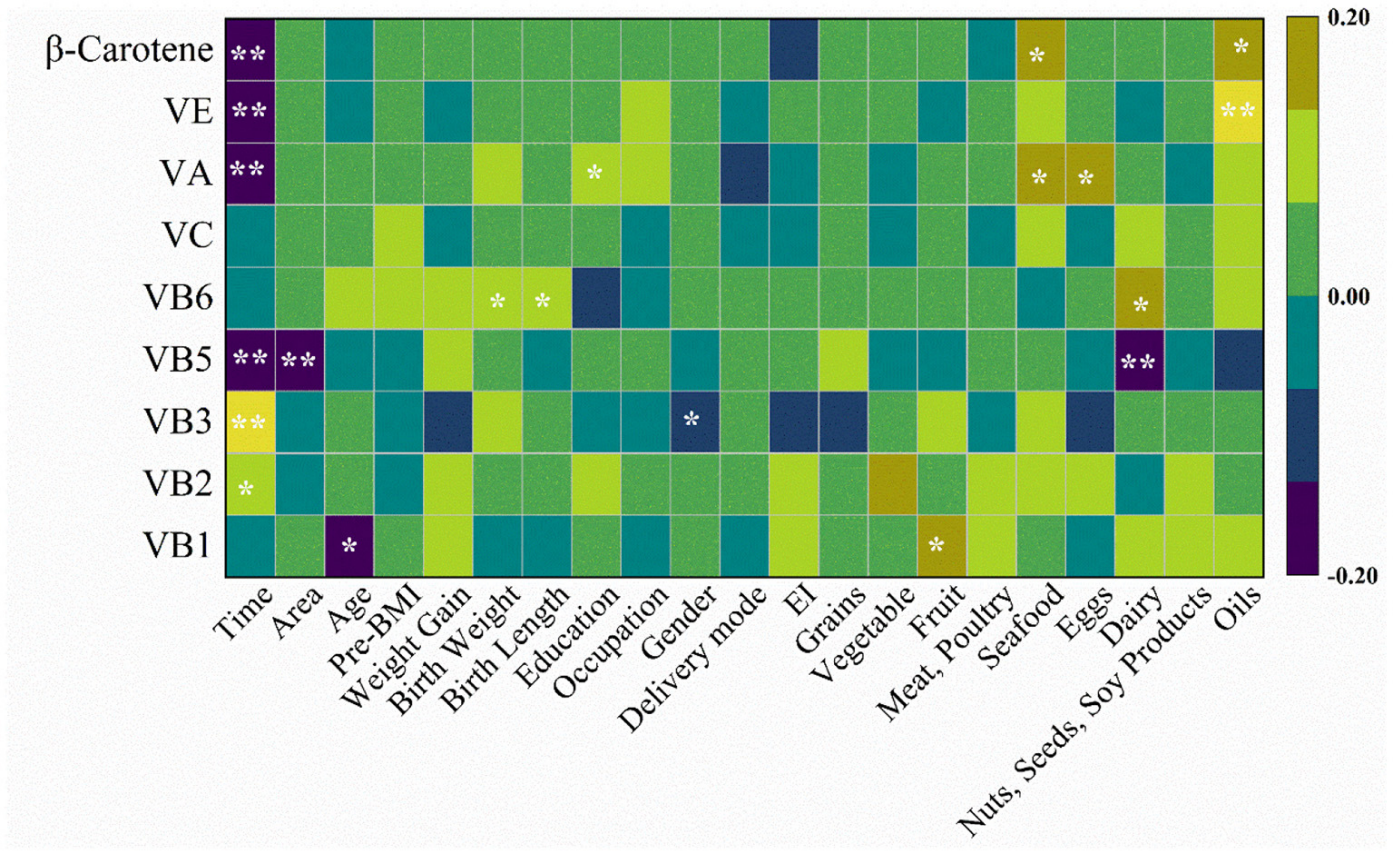
Heatmap displays correlation between HM vitamins and maternal-infant characteristics.

The correlation between HM vitamins contents and maternal–infant factors was revealed as shown in Figure 3. The exogenous factors (lactation period, region and diet records) were more correlated with the presence of HM vitamins contents, compared to the other three factors. Lactation period was significantly negatively correlated (| r | > 0.3) with pantothenic acid, vitamin A, and β-carotene. It also had a significant correlation with vitamin E (r = −0.196, | r | <0.3; *p* < 0.001), riboflavin (r = 0.129; *p* < 0.05) and niacin (r = 0.216; *p* < 0.001). There was a correlation between region and pantothenic acid (r = −0.16, *p* = 0.003). Dietary intake of mothers that showed correlation with HM vitamins during lactation included: the fruit was correlated with thiamine (r = 0.173, *p* = 0.016); the sea food was respectively correlated with vitamin A (r = 0.143, *p* = 0.048) and β-carotene (r = 0.146, *p* = 0.043); the eggs was correlated with vitamin A (r = 0.151, *p* = 0.037); the dairy was respectively correlated with pantothenic acid (r = −0.187, *p* = 0.009) and vitamin B_6_ (r = 0.149, *p* = 0.039); the oils was respectively correlated with vitamin E (r = 0.232, *p* = 0.001) and β-carotene (r = 0.174, *p* = 0.016). There was a correlation between physiological factors (age, birth weight, and birth length) and HM vitamins (thiamine and vitamin B_6_). Maternal age was correlated with thiamine (r = −0.138, *p* = 0.012), and vitamin B_6_ respectively had correlation with birth weight (r = 0.127, *p* = 0.02) and birth length (r = 0.108, *p* = 0.04). The general demographic factor (education) was correlated with vitamin A (r = 0.117, *p* = 0.033). The endogenous factor (infant gender) showed correlation (*p* = 0.026, *p* < 0.05) with niacin, and the point biserial correlation coefficient was −0.122.

### Multiple linear regression analysis

In order to explore the causation between HM vitamins and influence factors, the HM vitamins were used as regression dependent variables and statistically significant (| r | > 0.3, *p* < 0.05) influencing factors were introduced as regression independent variables according to the correlation analysis. The results of the equation models (vitamin A, β-carotene and pantothenic acid) were statistically significant by multiple linear regression analysis, as shown in Table 2. The variance inflation factors (VIF) was <5, which meant no multicollinearity between variables. Durbin–Watson (D–W) diagnosis proved no serial correlation. Thus, the regression models were effective. Lactation period (time) had explained the negative variation of vitamin A (21.2%), β-carotene (10.5%), and pantothenic acid (9.5%). The regression equation of vitamin A was y = 74.984−8.733 × X1, the adjustment R^2^ = 0.212. The regression equation of β-carotene was y = 5.712−0.795 × X1 + 0.048 × X2, the adjustment R^2^ = 0.118. The two variables (lactation period and oils) jointly had explained 11.8 % of the HM β-carotene variation. The regression equation of pantothenic acid was y = 294.416−19.454 × X1, the adjustment R^2^ = 0.095.

**Table 2:** Associations between mother-infant factors and HM vitamins according to multiple linear regression analyses.

		**B**	**Beta**	***t***	***p***	**VIF**	**R^2^**	**D–W**
**Vitamin A**	Constant	74.984		13.763	<0.001	1.000	0.212	1.741
	Time	−8.733	−0.465	−7.215	<0.001			
**β-carotene**	Constant	5.712		8.109	<0.001	1.000		1.639
	Time	−0.795	−0.347	−5.069	<0.001		0.105	
	Olis	0.048	0.135	1.976	0.048		0.118	
**Pantothenic acid**	Constant	294.416		15.378	<0.001	1.000	0.095	1.887
	Time	−19.454	−0.315	−4.598	<0.001			

### Discriminatory analysis

**Figure 4:**
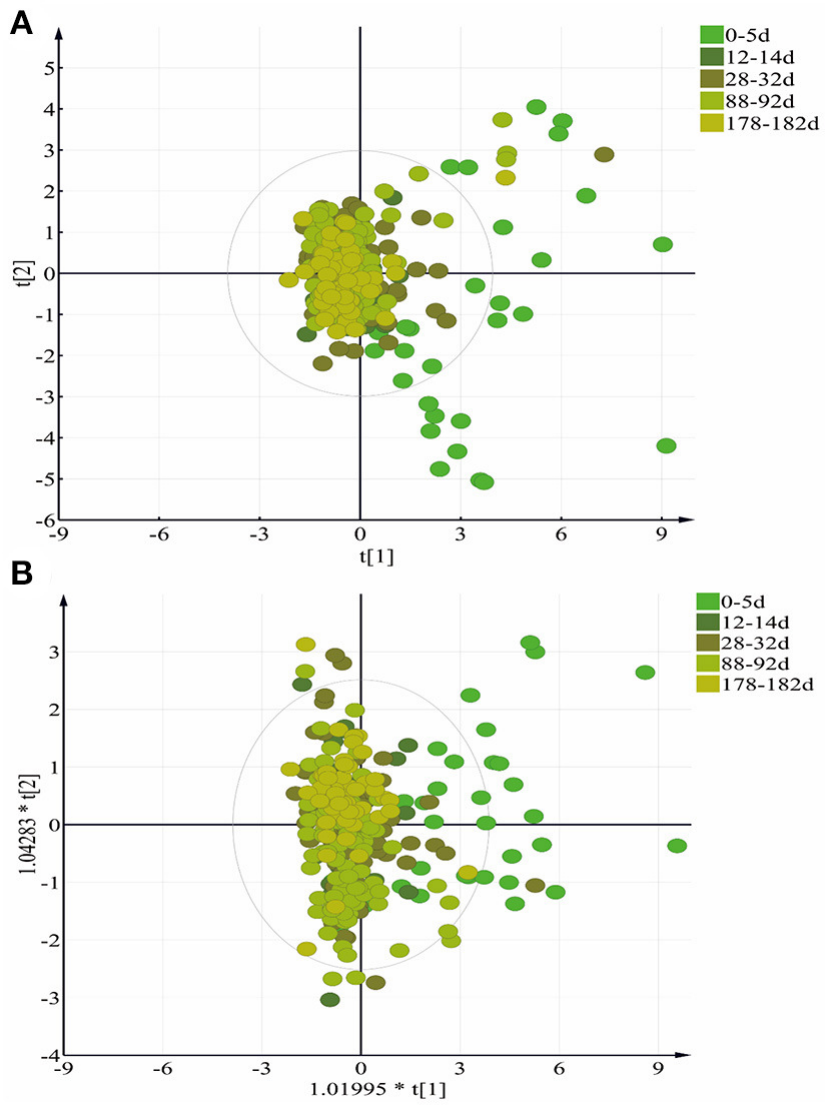
**(A)** Score plots for HM vitamin collected in different lactation period based on PCA model. **(B)** score plots for HM vitamin collected in different lactation period based on OPLS-DA mod.

HM vitamins were subjected to principal component analysis (PCA) modeling at different lactation stages colostrum (0–5 days), transition milk (12–14 days), and mature milk (28–32 days, 88–92 days, and 178–182 days). The contribution of the first two principal components to the variability of the data was 44.9%. As shown in Figure 4A, all HM samples were well clustered in the center, indicating good stability and accuracy of the characterization of HM vitamin components. However, for the 332 individual milk samples, many samples were mixed together. Based on the results of the PCA, the data were analyzed again using the orthogonal partial least squares discriminant analysis (OPLS-DA) model. Compared with PCA, OPLS-DA is a sophisticated supervised clustering method that generates a more appropriate separation of classes algorithm. The OPLS-DA model was constructed to reveal differences in vitamin content at different stages of lactation. The contribution of the first 4 components to the variability of the data was 61.4%. The score plot in Figure 4B shows the separation between the different lactation stages of the HM samples, and for the 332 milk samples, many samples overlapped, indicating that the HM vitamin components became stable during these lactation stages.

## Discussion

Previous publications (8, 13, 17) had adopted several methods for determining vitamins in human milk, for example, several HPLC–UV/FD and LC–MS/MS methods. HPLC method required complex sample pretreatment was commonly used for the determination of vitamins in the human milk. However, some water-soluble B-vitamin needed to be tested by the time-consuming microbiological method (12), for example vitamin B_6_. The reason was that the extremely low level of vitamin B_6_ in HM caused the natural fluorescence of vitamin B_6_ to be undetectable by the UV light detector of HPLC. Thus, the range of water–soluble vitamins contents using LC-MS/MS: thionine (0.002−0.221mg L^−1^), riboflavin (0.057−0.845 mg L^−1^), niacin (0.002−3.179 mg L^−1^), pantothenic acid (2.0−2.9 mg L^−1^), vitamin B6 (0.006−0.692 mg L^−1^), and vitamin C (0.11−64 mg L^−1^). This study had described those six water–soluble vitamins in HM could be quantified with a satisfactory chromatographic or MS resolution by LC–MS/MS. Even with identical product ions, no cross–talk occurred between the selected mass transitions (13). LC–MS/MS simplified the pre–treatment process, shortened the test time, and enabled high throughput required for a large number of samples.

Compared with other studies, the contents of the water–soluble vitamins were higher than in Japan (15), the United Kingdom (14), Cambodia (18), and other Chinese studies (8), except for vitamin C. The cause of low vitamin C levels mainly might be the different detection methods. Vitamin C, also known as ascorbic acid, was a polyhydroxy compound with acidic properties. The pretreatment method used in this paper might lead to a neutralization reaction between ascorbic acid and sodium hydroxide, which might result in lower results for ascorbic acid content. The other reason was that according to some traditional Chinese dietary habits, many mothers exclude most fruits and vegetables from the diet for the whole lactation after parturition, resulting in a major drop in their vitamin C. Therefore, this method of water–soluble vitamin was suitable for testing the other five B vitamins except vitamin C.

Most studies (6, 12) on water–soluble vitamins had focused on functional activities, relevant physical and chemical properties. For example, thiamin acted as a coenzyme for many important enzymes in the form of co–carboxylase, and niacin was involved in the metabolism activities (carbohydrate and fat) in the form of niacin and niacinamide. However, the special characteristics of HM samples and water–soluble vitamins (the low content, high variability, and diverse forms) in milk had led to fewer studies on water–soluble vitamins in breast milk. Published studies (1, 5, 18) had demonstrated that HM water–soluble vitamins were influenced by lactation period, area of residence, preterm birth, diurnal variation, education level, economic income, seasonal variation, nutritional status of nursing mothers, and dietary interventions. In the correlation analysis of this study, the lactation period had an effect on the water–soluble vitamin contents. All the water–soluble vitamins in colostrum were higher than in other stages, except riboflavin and niacin. Only niacin content increased with prolonged lactation, which was consistent with a published study (12). Age had a negative impact on the dynamics of thiamin. Fruit intake had a positive effect on thiamin content.

Infant gender had an effect on niacin content due to the involvement of niacin in the form of nicotinamide in important physiological activities of the body, such as genomic stability, neuroprotection and metabolism. Geographical distribution affected pantothenic acid content. The three cities (Beijing, Luoyang and Liuyang) are geographically progressively closer to the equator from north to south. The pantothenic acid content tended to decrease with the geographical distribution closer to the equator. The reason was due to the immune protective effect of breast milk on infants. With the increase of temperature and humidity, the human milk immune factor would produce an immune response to the outside world. Pantothenic acid as a coenzyme could participate in the body's metabolism, leading to a decrease in pantothenic acid content. However, no literature was available to explain the negative effect of milk intake on pantothenic acid content. The reason might be pantothenic acid was not only an anti–dermatitis factor, but also was involved in the synthesis of steroid hormones, vitamin A and vitamin D. Infant physiological factors (birth length and weight) had a significant positive effect on vitamin B_6_. Studies (6, 19) had confirmed that vitamin B_6_ played an important role in infant growth, immune function, anti–fatigue, and regulation of sterol hormone activity.

The correlation analysis in this paper demonstrated the relevance of maternal–infant factors on HM water–soluble vitamins. However, only pantothenic acid could be modeled as a causal factor by multiple regression analysis, and the significant influencing factor was lactation. The main physiological function of pantothenic acid is the formation of coenzyme A and acyl carrier protein (ACP), through which it exerts its metabolic effects. Several studies (6, 8) confirmed that the mammary gland lacked the ability to synthesize water–soluble vitamins and that water–soluble vitamins in milk were mainly derived from the maternal blood. Meanwhile, the mammary gland also lacked the ability to actively transport and metabolize water–soluble vitamins. Some literatures (7, 12) confirmed that oral supplementation with thiamine could not increase the water–soluble vitamin content in milk, but increased the thiamine content in maternal urine.

The range of fat–soluble vitamins contents in human milk were: vitamin A (40−64.6 μg 100mL^−1^), β-carotene (16−20.8 μg 100mL^−1^), and vitamin E (84−3404 μg 100mL^−1^) (20). Optimization of the sampling process, transportation process, and testing methods had contributed to higher HM vitamins contents than in other countries. Compared with other studies, the contents of the fat–soluble vitamins were higher than in Japan (15), the United Kingdom (14), Cambodia (18), Bangladesh (7), and other Chinese studies (8). Fat–soluble vitamins composed mainly of chemical elements (carbon, hydrogen, and oxygen) cannot provide energy to the body. Studies (8, 19, 21) had confirmed that factors affecting the content of HM fat–soluble vitamins included lactation period, age, number of births, duration of pregnancy, season, and education level. Correlation analysis in this paper confirmed that lactation showed a significant negative correlation for vitamin A and β-carotene and a negative effect for vitamin E. Fat–soluble vitamins were highest in colostrum, in agreement with the studies of China (8) and Tanzania (22). A study (23) had confirmed that fat–soluble vitamins interact with each other and that without dietary intervention, fat–soluble vitamins would remain in balance.

Vitamin A was oxidized in the body then converted to retinoic acid. Retinoic acid was an important form of vitamin A that exerted a variety of biological actions, such as maintaining epithelial cell activity, regulating lymphocyte function, and mediating cellular bioactivity. Carotenoids, such as β-carotene, were known as precursors for the synthesis of vitamin A. They were partially converted to vitamin A and a potential source of vitamin A for breastfed infants. β-carotene, which accounted for 25% of total carotenoids, could be converted to vitamin A, also known as pro–vitamin A. In addition to synthetic vitamin A, β-carotene has immunoprotective and antioxidant properties and other health effects. The mother could pass vitamin E to the fetus through the placenta during pregnancy. For infants who were exclusively breastfed, vitamin E from milk was the only source. It was a hormone precursor with a steroidal structure. Vitamin E was also known as tocopherol. Vitamin E in nature consisted of two groups, tocopherols, and tocotrienols. 28% of vitamin E in HM was in the form of alpha–tocopherol. In this paper, no dietary intervention was imposed on the subjects, so that diet had a positive effect on fat–soluble vitamins. The vitamin A and β-carotene could be modeled as the causal factor by multiple regression analysis, and the largest influencing factor was lactation. Vitamin A and β-carotene showed a significant negative correlation with lactation period. Although many maternal–infant factors were correlated with HM vitamins contents in the correlation analysis. However, when the maternal–infant factors as independent variables unsatisfied the condition (| r | > 0.3, *p* < 0.05), no multiple linear regression can be performed. The independent variable would be excluded from the regression model.

## Conclusions

In order to optimize the HM vitamins, this work had explored the influence of maternal–infant factors on the HM vitamins dynamics through Chinese 147 mother–infant pairs cohort. The average HM vitamins (0–6 months) were thiamine (8.07 ± 5.59 μg 100g^−1^), riboflavin (91.22 ± 28.89 μg 100g^−1^), niacin (78.96 ± 53.43 μg 100g^−1^), pantothenic acid (221.68 ± 121.45 μg 100g^−1^), vitamin B6 (6.59 ± 2.67 μg 100g^−1^), vitamin C (634.23 ± 537.50 μg 100g^−1^), vitamin A (39.36 ± 34.13 μg 100g^−1^), β-carotene (6.21 ± 3.05 μg 100g^−1^) and vitamin E (354.85 ± 269.68 μg 100g^−1^). The three vitamins (vitamin A, β-carotene, and pantothenic acid) showed significant downward trends with lactation period (| r | > 0.3, *p* < 0.05). The lactation period factor could explain the negative variation of vitamin A (21.2%) and pantothenic acid (9.5%). The factors of lactation period and oils intake could jointly explain variations of β-carotene (11.8%). The results showed that exogenous factors (lactation and dietary intake) could explain the dynamics of vitamin content in human milk, including vitamin A, pantothenic acid and β-carotene. The aim of this study was to find mother-infant factors affecting the dynamics of HM vitamins in order to optimize HM vitamins and Improve the long- and short-term health of infants.

## Data availability statement

The original contributions presented in the study are included in the article/supplementary materials, further inquiries can be directed to the corresponding author.

## Ethics statement

The studies involving human participants were reviewed and approved by NCT02658500. Written informed consent to participate in this study was provided by the participants' legal guardian/next of kin.

## Author contributions

All authors listed have made a substantial, direct, and intellectual contribution to the work and approved it for publication.

## Funding

The main funding for the study came from the National Natural Science Foundation of China (Grant No. 32072191), Daxing District Major Scientific and Technological Achievements Transformation Project (Grant No. 2020006), Beijing Science and Technology Plan (Grant No. Z201100002620005), and Guangxi Science and Technology Project (AD20297088).

## Conflict of interest

Authors WQ, JC, MZ, YW, JZ, BY, and LC were employed by the company Beijing Sanyuan Foods Co., Ltd. The remaining author declares that the research was conducted in the absence of any commercial or financial relationships that could be construed as a potential conflict of interest. The authors declare that this study received funding from Beijing Sanyuan Foods Co., Ltd. The funder had the following involvement in the study: Maternal and Infant Nutriomics Cohort Study.

## Publisher's note

All claims expressed in this article are solely those of the authors and do not necessarily represent those of their affiliated organizations, or those of the publisher, the editors and the reviewers. Any product that may be evaluated in this article, or claim that may be made by its manufacturer, is not guaranteed or endorsed by the publisher.
